# Seronegative Infection with *Toxoplasma gondii* in Asymptomatic Human Immunodeficiency Virus Type 1 (HIV-1)-Infected Patients and in Blood Donors

**DOI:** 10.3390/jcm11030638

**Published:** 2022-01-27

**Authors:** Agnieszka Pawełczyk, Małgorzata Bednarska, Kamila Caraballo Cortés, Marianna Glamkowska-Sady, Justyna Kowalska, Beata Uszyńska-Kałuża, Marek Radkowski, Renata Welc-Falęciak

**Affiliations:** 1 Department of Immunopathology of Infectious and Parasitic Diseases, Medical University of Warsaw, ul. Pawińskiego 3C, 02-106 Warsaw, Poland; kcaraballo@wum.edu.pl (K.C.C.); mariannags@interia.pl (M.G.-S.); marek.radkowski@wum.edu.pl (M.R.); 2 Department of Parasitology, Faculty of Biology, University of Warsaw, ul. Miecznikowa 1, 02-096 Warsaw, Poland; mabed@biol.uw.edu.pl (M.B.); rwelc@biol.uw.edu.pl (R.W.-F.); 3 Department of Adults’ Infectious Diseases, Medical University of Warsaw, ul. Wolska 37, 01-201 Warsaw, Poland; justyna.kowalska@wum.edu.pl; 4 Blood Center, Ministry of Internal Affairs and Administration, ul. Wołoska 137, 02-507 Warsaw, Poland; beausz@wp.pl

**Keywords:** toxoplasmosis, *Toxoplasma gondii*, HIV/AIDS infection, blood donors, seroprevalence, seronegative infection, DNA identification

## Abstract

Background: Toxoplasmosis is a common opportunistic infection in AIDS patients. The routine diagnostics is based on serologic testing and IgG avidity index, but it may have limited utility in immunodeficient patients; thus, it is recommendable to detect *T. gondii* DNA in subjects with advanced HIV disease. The results of the studies published so far focused on patients with clinical symptoms of toxoplasmosis. Our study encompassed a group of HIV-infected subjects on cART therapy, without immunological disturbances and clinical symptoms of *T. gondii* infection. Methods: The study was retrospective, and samples were collected between 2013 and 2016. We evaluate the prevalence of serological (IgM, IgG, and avidity IgG) and molecular (DNA) *T. gondii* infection markers in asymptomatic HIV-infected patients and the control group using serologic (ELISA) and quantitative (real-time PCR) molecular testing. Results: Of 152 HIV-infected in routine follow-up tested for *T. gondii* IgM and IgG, 6 (3.9%) and 50 (32.9%) were positive, respectively. Of 168 serum samples from blood donors, 1 (0.6%) and 49 (29.2%) were IgM^+^ and IgG^+^ positive, respectively. IgM seroprevalence in HIV-infected patients was significantly higher than in blood donors. *T. gondii* DNA (genotype II) was identified in 47 (30.9%) HIV-infected patients, with 13 (8.6%) IgM^−^IgG^−^ samples. In blood donors, *T. gondii* DNA was present in 15 (8.9%) IgM^−^IgG^−^. Conclusions: In both groups, *T. gondii* DNA was detectable in seronegative subjects, implying the need to supplement the routine serological testing via the molecular method. It can help the accurate monitoring of the reactivation of infection in asymptomatic HIV-infected persons, and the quick introduction of specific therapy, in blood donors, would be of high importance for safe blood donations.

## 1. Introduction

*Toxoplasma gondii* is an intracellular parasite, the causative agent of toxoplasmosis. Epidemiological data indicate that the global incidence of *T. gondii* infections is about 30%, varying depending on the geographical region, from 1% to over 96% [1,2,3]. Most primarily infected subjects develop a subclinical form of parasitosis, resulting in a latent infection [4,5]. Importantly, in the acute phase of *T. gondii* infection, tachyzoites, are present in blood and may cause complications to potential blood or graft recipients, including a severe course of toxoplasmosis, due to comorbidities and/or immune suppressive treatment [5]. Unfortunately, donated blood in Poland is not routinely tested for *T. gondii* infection, and there are no clear guidelines, regarding blood donation qualification during the active infection [6].

Toxoplasmosis is one of the most common opportunistic diseases observed in the course of HIV/AIDS, and the leading cause of mortality in this group of patients worldwide [5,7]. It is estimated that the frequency of HIV/*T. gondii* co-infection is more than 50% [8,9,10], and reactivation of latent infection is typically responsible for toxoplasmosis of the central nervous system (TCNS), which may have fatal consequences [9]. The risk of developing TCNS is estimated to be from 30% to 40% in untreated HIV/AIDS patients with a CD4^+^ T-cell count lower than 100 cells per mm^3^ [2,11,12,13,14,15].

The routine diagnostic of toxoplasmosis is based on serologic tests (detection IgM/IgA/IgG and assessment of IgG avidity index); however, they may have a low predictive value, especially in patients with HIV-related severe immune deficits (atypical serologic window or a seronegative course of infection) [8,16]. Available research and diagnostic studies on *T. gondii* infection are mainly focused on assessing the IgG avidity index, in the context of vertical infection, rather than in immunocompromised host or blood donors [16,17,18].

Detection of IgG antibodies in clinically asymptomatic *T. gondii* infection cannot distinguish between the latent or past infection and should be complemented with the detection of parasitic DNA [19,20]. On the other hand, isolated detection of *T. gondii* DNA without serologic testing does not allow to differentiate between active and latent infection [19,20].

In HIV-infected patients with toxoplasmosis reactivation, the presence of *T. gondii* antibodies is usually accompanied by parasitic genetic material [5,20,21]; thus, epidemiological control of the *T. gondii* infection in these patients requires systematic monitoring of both IgM and IgG antibodies, as well as *T. gondii* DNA.

The acute diagnostics of *T. gondii* infection in HIV patients requires adequate information, regarding the prevalence of *T. gondii* antibodies and DNA, in general different populations. Due to limited data on molecular diagnosis of the *Toxoplasma* infection and its association to *T. gondii* antibodies in HIV patients in Poland, we aimed to determine the prevalence of antibodies and DNA *T. gondii*. The specifical aim of the study was to assess the incidence of *T. gondii* infection in toxoplasmosis-asymptomatic HIV-infected patients under antiretroviral treatment, as well as in blood donors using both serologic and molecular testing [17,22,23]. Sequencing of positive samples revealed that all of them were closely related (<99%) to isolates of genotype II *T. gondii*. The sequence was deposited in the GenBank NCBI database, under the accession number OK338428.

The first study of this type in Poland provides evidence that, in both studied groups, *T. gondii* DNA was accompanied by IgM and/or IgG antibodies but was often detectable in the absence of any of these serologic markers, which may indicate a seronegative course of infection.

## 2. Materials and Methods

### 2.1. Study Groups

Serum samples and whole blood were collected, in years 2013–2016, from 152 HIV-1-infected patients who routinely followed up at the HIV Outpatients’ Clinic of the Hospital for Infectious Diseases in Warsaw, as well as from 168 blood donors who represented a control group (Table 1). The subjects were screened for *T. gondii* infection in AmerLab Ltd. Diagnostic Laboratory of Parasitic Diseases and Zoonotic Infections in Warsaw. Medical and epidemiological characteristics included age, sex, lymphocyte CD4^+^ T cell count, plasma HIV RNA level, and cART therapy (Table 1). No clinical symptoms of toxoplasmosis were observed in the group. In HIV-infected patients, no immune disturbances were found.

### 2.2. Study Design

The study was retrospective. All samples were analyzed two times; in the case of discrepancies, a third testing was performed.

#### 2.2.1. Serologic Tests

IgM and IgG *T. gondii* antibodies were determined in all serum samples. IgG avidity was determined only for IgG-positive samples. The presence of *T. gondii* antibodies was determined using NovaTec Immunodiagnostica GmbH-Test NovaLisa *T. gondii* IgM µ-capture (Frankfurt, Germany) and NovaTec Immunodiagnostica GmbH-Test NovaLisa *T. gondii* IgG (Frankfurt, Germany), as well as the NovaTec Immunodiagnostica GmbH-Test NovaLisa^®^ Avidity *T. gondii* IgG (Frankfurt, Germany) test, according to the manufacturer’s instructions and interpretation criteria.

#### 2.2.2. DNA Isolation, Real-Time PCR, and Sequencing

Genomic DNA was isolated from whole blood using the DNeasy Blood and Tissue kit (Qiagen, Crawley, UK). Detection of *T. gondii* was performed by real-time PCR amplification of 115 bp sequence from B1, a 35-fold repetitive gene comprising of 2214 nucleotides in each repeat, which is highly conserved among *Toxoplasma* strains [24]. Real-time PCR (quantitative) was performed using all samples. Oligonucleotides and the thermal profile of amplification used in this study have been described [25]. DNA extracted from tachyzoites of *T. gondii* RH strain from cell culture was used as a positive control. In all positive samples obtained in real-time PCR, conventional PCR was used for the amplification of a non-coding 529 bp DNA fragment B1 gene, repeated 200 ± 300 times in the *T. gondii* genome. The primers and thermal profile of conventional PCR amplification used in this study have been described [26]. Sequencing of positive samples revealed that all of them were closely related (<99%) to the isolates of genotype II *T. gondii*. The sequence was deposited in the GenBank NCBI database under the accession number OK338428.

### 2.3. Statistical Analysis

Statistical analysis was performed using the IBM SPSS Statistics v. 25.0 software (IBM, Armonk, NY, USA). The equivocal serologic results of *T. gondii* IgM and IgG detection were classified as negative. A descriptive analysis of the study participants was included, and calculations of seroprevalence rates were performed. Seroprevalence rates, age, and sex, were compared between the tested groups (HIV–infected patients/blood donors), and seroprevalence rates and lymphocyte T CD4^+^ count were also compared, within HIV-infected patients, using maximum likelihood techniques based on log-linear analysis of contingency tables (HILOGLINEAR).

### 2.4. Ethical Approval and Consent to Participate

The study protocol followed the ethical guidelines of the 2013 Declaration of Helsinki and was approved by the Internal Review Board of the Warsaw Medical University (consent number AKBE/43/20).

## 3. Results

### 3.1. T. gondii IgM and IgG Seroprevalence

Table 2 shows that of the 152 HIV-infected patients tested for *T. gondii*-specific IgM and IgG, 6 (3.9%) and 50 (32.9) were positive, respectively. Among 168 serum samples, collected from blood donors, 1 (0.6%) and 49 (29.2%) were positive for IgM and IgG, respectively.

IgM seroprevalence in the HIV group was significantly higher, when compared to blood donors (*p*  =  0.03).

Three out of six IgM-positive HIV patients probably displayed early infection (detection of exclusively IgM or IgM, as well as low avidity IgG) (Table 2 and Table 3).

Another IgM-positive patient revealed high avidity IgG fulfilling criteria of subacute infection, infection reactivation, or persistent IgM. In the remaining two cases, inconclusive results were the detection of IgM, as well as equivocal avidity IgG (Novatec unit test) (Villard 2016). Results defined as “inconclusive results” do not allow us to unequivocally determine whether the result is positive or negative. The range of values obtained is within the so-called “grey zone”, defined by the test manufacturer. According to the manufacturer’s guidelines, in case of inconclusive results, it is recommended to retest in a sample collected after 2 to 4 weeks.

In the blood donor group, IgM antibodies were found in one (0.6%) case in the presence of high avidity IgG antibodies, which indicates subacute infection, reactivation of the infection, or persistent IgM.

### 3.2. Avidity of T. gondii IgG

All anti-*T. gondii* IgG-positive samples were tested for antibody avidity. In HIV-infected patients, low avidity index (<45%) was observed in 3 cases (avidity values: 11.8%, 28.7%, and 34%), equivocal avidity (45–60%) in 4 (values: 45.1%, 51.7%, 57.9%, and 58.2%), while in the remaining 43 samples, the avidity values were high (>63.2%) (Table 2). High avidity (>65%) was found in all 49 IgG-positive samples from blood donors (Table 2).

### 3.3. Detection of T. gondii DNA

*T. gondii* DNA was identified in 47 (30.9%) of HIV-infected patients and 29 (17.3%) of blood donors (Table 2) (*p* < 0.01). All isolated were closely related (<99%) to the isolates of genotype II, which is the most common in Europe.

In HIV-infected patients, *T. gondii* DNA prevalence increased with age, 0% (<25 years), 46.2% (25–35 years), and 53.8% (36–51 years), while, in blood donors, it fluctuated, 24.1% (<25 years), 20.7% (25–35 years), and 55.2% (36–54 years); however, none of these differences were statistically significant. No correlation was found between *T. gondii* DNA and lymphocyte CD4^+^ T cell count, HIV RNA, or HAART.

In the HIV group, *T. gondii* DNA was detected in 2 (1.3%) and 28 (18.4%) cases reactive for IgM or IgG antibodies, respectively, and in 4 (2.6%) subjects positive for IgM and IgG antibodies (Table 2 and Table 3). The IgG avidity index in *T. gondii* DNA-positive samples ranged from 11.8% to 62.3%. Furthermore, we identified *T. gondii* DNA in 13 (8.6%) patients, in the absence of both IgM and IgG antibodies.

Among the blood donors group, 14 (8.3%) subjects positive for *T. gondii* DNA also revealed detectable *T. gondii* antibodies, 1 (0.6%) IgM-positive and 13 (7.7%) IgG-positive, while 15 (8.9%) subjects were positive for *T. gondii* DNA of neither the IgM nor IgG antibodies (Table 2). The value of the IgG avidity index in all DNA-positive cases was above 60% (Table 2).

This section may be divided into subheadings. It should provide a concise and precise description of the experimental results and their interpretation, as well as the experimental conclusions that can be drawn.

## 4. Discussion

The presented study aimed to assess the frequency of anti-*T. gondii*-specific antibodies and parasitic DNA among HIV-1-infected patients followed at outpatients clinic for toxoplasmosis, when compared to control group of healthy blood donors.

The severe clinical consequences of toxoplasmosis in HIV-1-infected subjects with severe immunosuppression, CD4 cell count <100 cells/mm^3^, requires the precise monitoring of the *T. gondii* infection markers [9,27].

On the other hand, typically, the asymptomatic course of toxoplasmosis in blood donors, along with limited clinical data available for this group, creates an underestimated risk of *T. gondii* infection in blood recipients [28]. Transmission of *T. gondii* by whole blood transfusions, white blood cell transfusions [29,30,31,32], or organ transplantation [33,34,35] from seropositive donors to susceptible recipients is possible [28]. Thus, for both groups, it is crucial to implement diagnostics that would unambiguously identify an active, past infection or reinfection [6]. Furthermore, due to the possible seronegative course of the *T. gondii* infection (e.g., in immunocompromised blood recipients or HIV-1 co-infected patients [28,36,37,38]), special attention should be paid to the importance of the detection of parasitic genetic material [5,12].

Assessment of *T. gondii* seroprevalence in HIV-1 positive patients revealed significant differences among geographical areas (from 8–44% in Japan, East China, and India to 62–94% in Morocco and Ethiopia) [7,27,28,37,39,40,41]. In our study, the prevalence of *T. gondii* antibodies among HIV-1-infected patients was 36.8%, which was similar to the results obtained in other European countries (23.04% in France, 34.7% in Spain, 35.5% in Germany, and 36.8% in Italy) [39,42]. In Poland, the seroprevalence of the *T. gondii* infection in AIDS patients was estimated to be 5.4%, but these data were limited to symptomatic toxoplasmosis [14].

In this study, we did not find statistically significant differences between the seroprevalence of the *T. gondii* infection among HIV-1 positive patients and the control group of blood donors, probably due to effective cART therapy in the majority (73%) of HIV-1-infected patients and, consequently, neither significant immune system disorders nor overt symptoms of toxoplasmosis. However, these patients should be still considered to be at risk of a severe course of the *T. gondii* infection.

The prevalence of anti-*T. gondii* antibodies among blood donors in Poland (29.8%) observed in our study was lower than that observed worldwide (34% in the Czech Republic to 80% in African and Asian countries) [6,42,43], which could be explained by the differences in the effectiveness of parasite transmission in local human populations [44].

Differentiation of the class of detected antibodies and assessment of IgG avidity allows us to estimate the risk of clinically overt toxoplasmosis in HIV-infected patients, while the control group is of high importance, in the context of safe blood donation. Detection of exclusively IgM antibodies (IgM^+^IgG^−^) is an indicator of early infection. Detection of IgM and low avidity IgG (IgM^+^IgG^+^low) indicates active infection, while IgM is combined with high avidity IgG (IgM^+^IgG^+^high), subacute infection, reactivation of the infection, or persistent IgM (detectable up to 2 years after primary infection). In contrast, detection of exclusively high avidity IgG antibodies (IgM^−^IgG^+^high) characterizes past toxoplasmosis, while the detection of exclusively low avidity IgG antibodies (IgM^−^IgG^+^low) characterizes chronic infection or subacute infection [13,16,28].

In our study, there were differences in the *T. gondii* serologic profile, both within the studied groups and between the groups. In both groups, IgM antibodies were found less frequently than IgG antibodies. IgM was detected significantly more frequently in HIV-infected patients (3.9%) than in blood donors (0.6%). In other studies, the estimated prevalence of anti-*T. gondii* IgM antibodies in HIV-infected subjects varied from 1.2% to 4.9% [5,23,39].

In the group of blood donors, IgM was found in one subject in the presence of high avidity IgG antibodies; whereas, in the remaining cases, exclusively high-avidity IgG was detected [5,6,20]. For example, in a study conducted in Iran, high avidity IgG was detected in 81% of subjects, while low and equivocal avidity were found in 11.26% and 7.74% of cases, respectively [20]. Similarly, in India, the majority (96.3%) of blood donors displayed high avidity IgG, while only 3.7% displayed low avidity [5]. Unfortunately, the number of studies assessing the avidity level of IgG in blood donors in Europe is limited, since the available research was mostly focused on the aspect of vertical transmission [16].

In the active phase of the *T. gondii* infection, tachyzoites may be transmitted to the new host, e.g., during a blood transfusion or transplantation, which may pose severe complications in a potential recipient [5,11,16]. The isolated detection of IgG antibodies has limited utility in the characterization of the infection phase in a blood or transplant donor. Unfortunately, in Poland, there are no clear guidelines regarding the qualification of blood donors based on the markers of the *T. gondii* infection, while, e.g., in France, diagnostic recommendations have been developed and depend largely on a specific group (blood donors, pregnant women, or immunocompromised hosts) [16,45,46].

Given a relatively low predictive value of serologic tests in patients with immune disorders, including HIV-infection with CD4^+^ count <100 cells/mm^3^ and molecular testing characterized by higher sensitivity and specificity, is proposed [16,36,40,46]. Currently, according to the recommendations of the Polish AIDS Society, all symptomatic patients should be tested with PCR for the presence of *T. gondii* DNA in cerebrospinal fluid http://www.ptnaids.pl/index.php?option=com_content&view=article&id=179%3A-wytyczne-ptn-aids-2021, accessed on 12 December 2021. Testing patients’ blood samples for *T. gondii* DNA could be of more applicable use. Our results revealed the presence of *T. gondii* DNA in 47 (30.9%) of HIV-infected patients, including 34 (22.3%) of seropositive infections, which is comparable to the studies of Rostami et al. and Colombo [12,19]. However, in 8.6% of cases, none of the antibodies were detected, which may indicate either a low antibody level (below the sensitivity limit of detection) or a seronegative course of infection [18] and justify the use of molecular testing for detecting *T. gondii* DNA. Sequencing of positive samples revealed that all of them were closely related (<99%) to isolates of genotype II *T. gondii*, which is the most common in Europe [5,39,47]. The sequence was deposited in the GenBank NCBI database under the accession number OK338428.

HIV-infected patients with a CD4^+^ lymphocyte count below 100 cells/mm^3^ present a higher risk of clinically overt opportunistic infections, including *T. gondii*-induced encephalitis and ocular toxoplasmosis [15,24,36].

Some authors suggest that the detection of *T. gondii* DNA always indicates active toxoplasmosis in HIV-infected patients and is reflected by clinical manifestations [21]. This opinion was not supported in our study, since clinical manifestations of toxoplasmosis were not observed. Furthermore, neither a correlation between *T. gondii* DNA and CD4 T-cell count nor between *T. gondii* DNA and HIV viral load was found. Gashout et al. detected *T. gondii* DNA in 72.1% of seropositive, asymptomatic HIV-infected patients with a CD4 T-cell count below 100 cells/mm^3^ [15,21]. In a similar study, *T. gondii* DNA was detected in 54.7% of *T. gondii* seropositive HIV-infected patients but significantly more often in patients with lower a CD4 count (<100 cells/mm^3^) [21,22].

In blood donors, *T. gondii* DNA was found in 29 samples (17.3%), including 1 (0.6%) IgM- and 13 (7.7%) IgG-positive, while no antibodies were detected in 15 (8.9%) samples. These results were not similar to HIV-infected patients, probably reflecting that an intact immune system condition among HIV-infected patients is most likely due to ongoing cART. However, the presence of *T. gondii* DNA in a relatively high proportion of seronegative samples from blood donors may imply seronegative course toxoplasmosis. It raises blood safety issues because of the risk of transfusion-transmitted infection and justifies a need to introduce toxoplasmosis screening based on both serologic and molecular testing. Since the detection of *T. gondii* DNA in asymptomatic, seronegative donors does not distinguish between the active or latent form of toxoplasmosis, it justifies the search for new markers that would more accurately determine the phase of infection and estimate the risk of toxoplasmosis-like cell-free DNA.

## 5. Conclusions

*T. gondii* DNA was found in samples both with the presence of IgM and/or IgG antibodies, as well as with the absence of any serologic markers, indicating a seronegative course of infection among clinically asymptomatic HIV patients and blood donors. In HIV-infected subjects it advocates the need for a careful clinical follow-up and monitoring toxoplasmosis, using both serologic and molecular methods and, if necessary, quick introduction of a specific therapy. In blood donors, such combined monitoring would also be of high importance for safe blood donations.

## Figures and Tables

**Table 1:** Clinical and epidemiological characteristics of the studied groups.

	Median Age (Range) (Years)	Sex (F/M, (%))	Median Lymphocyte CD4^+^ Count Cells/mm^3^ (Range)	Median Plasma HIV RNA (Range) (Copies/mL)	HAART (%)
HIV+ (*n* = 152)	31 (20–51)	22/130 (14.5%/85.5%)	348 (110–9956)	21,262 (112–159,787)	111 (73%)
Blood donors (control group) (*n* = 168)	33 (18–54)	58/110 (34.5%/65.5%)	N/A	N/A	N/A

F—females; M—males; N/A—not available or applicable.

**Table 2:** *T. gondii* infection markers (IgM and IgG antibodies, IgG avidity index, and DNA) in the blood of HIV-infected patients and blood donors.

	*T. gondii* Antibody (Total)				*T. gondii* DNA Positive					*T. gondii* DNA Negative	
					*T. gondii* Antibody				Total	IgG Antibody	
	Positive		Avidity *	Negative	Positive			Negative		Positive	Negative
	IgM	IgG	IgG	IgM and IgG	IgM	IgG	IgM and IgG	IgM and IgG			
HIV+ (*n* = 152)	6 (3.9%)	50 (32.9%)	Low: 3 (6%) Equivocal: 4 (8%) High: 43 (86%)	96 (63.1%)	2 (1.3%)	28 (18.4%)	4 (2.6%)	13 (8.6%)	47 (30.9%)	22 (14.3%)	83 (54.6%)
Blood donors (*n* = 168)	1 (0.6%)	49 (29.2%)	High: 49 (100%)	118 (70.2%)	1 (0.6%)	13 (7.7%)	0	15 (8.9%)	29 (17.3%)	21 (12.5%)	118 (70.2%)

All samples were analyzed two times; in the case of discrepancies, a third testing was performed. * Avidity index interpretation (NovaTec): <45%—low, 45–60%—equivocal, >60%—high.

**Table 3:** Concomitant presence of IgM antibodies and *T. gondii* DNA among HIV-1-infected patients.

N° of Sample	Anti–*T. gondii*		Avidity *	*T. gondii* DNA	HIV RNA(Copies/mL)	CD4^+^ (Cell/mm^3^)	Most Probable Diagnosis
	IgM	IgG					
1	positive	positive	62.3% (high)	positive	25,849	324	subacute infection/reactivation or persistent IgM
2	positive	positive	51.5% (equivocal)	positive	474,875	100	inconclusive results
3	positive	positive	11.8% (low)	positive	1,012,778	124	primary infection
4	positive	negative	N/A	positive	6692	409	early stage of primary infection
5	positive	positive	48.7% (equivocal)	positive	nd	nd	inconclusive results
6	positive	negative	N/A	positive	nd	nd	early stage of primary infection

* Avidity index interpretation (NovaTec): <45%—low, 45–60%—equivocal, >60%—high. N/A—not available or applicable, nd—undetectable. Algorithm for the serologic result interpretation: IgM^+^ and low avidity. IgG—primary infection; IgM^+^ and high avidity IgG—subacute infection/reactivation or persistent IgM; IgM^−^ and low avidity IgG—chronic infection or subacute infection; IgM^−^ and high avidity IgG—past infection; IgM^+^ and equivocal avidity IgG—inconclusive results; IgM^+^ and IgG^−^—early stage of primary infection.

## Data Availability

The original data were limited, according to the Warsaw Medical University policy, which was not available outside the facility.
